# Depression treatment response to ketamine: sex-specific role of interleukin-8, but not other inflammatory markers

**DOI:** 10.1038/s41398-021-01268-z

**Published:** 2021-03-15

**Authors:** Jennifer L. Kruse, Megha M. Vasavada, Richard Olmstead, Gerhard Hellemann, Benjamin Wade, Elizabeth C. Breen, John O. Brooks, Eliza Congdon, Randall Espinoza, Katherine L. Narr, Michael R. Irwin

**Affiliations:** 1 Cousins Center for Psychoneuroimmunology, University of California at Los Angeles, Los Angeles, CA USA; 2 Jane and Terry Semel Institute for Neuroscience and Human Behavior at UCLA, Department of Psychiatry and Biobehavioral Sciences, David Geffen School of Medicine, Los Angeles, CA USA; 3 Department of Neurology, University of California at Los Angeles, Los Angeles, CA USA

**Keywords:** Predictive markers, Depression

## Abstract

Inflammation plays a role in depression pathophysiology and treatment response, with effects varying by sex and therapeutic modality. Lower levels of interleukin(IL)-8 predict depression response to antidepressant medication and to electroconvulsive therapy (ECT), although ECT effects are specific to females. Whether IL-8 predicts depression response to ketamine and in a sex-specific manner is not known. Here, depressed patients (*n* = 46; female, *n* = 17) received open label infusion of ketamine (0.5 mg/kg over 40 min; NCT02165449). Plasma levels of IL-8 were evaluated at baseline and post-treatment. Baseline levels of IL-8 had a trending association with response to ketamine, depending upon sex (responder status × sex interaction: *p* = 0.096), in which lower baseline levels of IL-8 in females (*p* = 0.095) but not males (*p* = 0.96) trended with treatment response. Change in levels of IL-8 from baseline to post-treatment differed significantly by responder status (defined as ≥50% reduction in Hamilton Depression Rating Scale [HAM-D] Score), depending upon sex (responder status × sex × time interaction: *F*(1,42)=6.68, *p* = 0.01). In addition, change in IL-8 interacted with sex to predict change in HAM-D score (β = -0.63, *p* = 0.003); increasing IL-8 was associated with decreasing HAM-D score in females (*p* = 0.08) whereas the inverse was found in males (*p* = 0.02). Other inflammatory markers (IL-6, IL-10, tumor necrosis factor-α, C-reactive protein) were explored with no significant relationships identified. Given these preliminary findings, further evaluation of sex differences in the relationship between IL-8 and treatment response is warranted to elucidate mechanisms of response and aid in the development of personalized approaches to depression treatment.

## Introduction

Depression is the leading worldwide cause of disability^1^, and females are impacted by depression at twice the rate of males^2^. Depression can be difficult to treat, with one third of patients failing to remit following multiple interventions^3^; this group is classified as suffering from treatment resistant depression (TRD). Unfortunately, there are no reliable clinical strategies for selecting an antidepressant treatment strategy more likely to lead to response. Elucidation of individual characteristics that predict and correlate with response to specific interventions is needed, directing clinicians toward treatments most likely to lead to response, while also providing information regarding potential underlying mechanisms.

Inflammation is implicated in both the pathophysiology and treatment responsiveness of depression^4–12^, and females appear to have differential behavioral and neural responses to inflammation^13,14^. Higher inflammation is associated with poorer response to most antidepressant strategies^15–19^, and thus may be of particular relevance in the study of TRD. Interestingly, while higher inflammation has been associated with poorer outcome to many pharmacologic treatments for depression, the inverse relationship has been found for treatment responsiveness to electroconvulsive therapy (ECT)^20,21^, an intervention used primarily for TRD or other severe mood disorders (e.g. catatonia, psychotic depression).

Ketamine is a rapidly acting treatment now extensively studied for TRD, though relatively little is known about the relation between inflammation and ketamine response. Based on pharmacologic properties and animal studies^22^, it is hypothesized that depressed patients with higher levels of inflammation may be more responsive to ketamine treatment^23^, as supported by some^24,25^ but not all treatment studies^26,27^. Though an animal study has demonstrated the benefits of ketamine for behavioral symptoms elicited by an inflammatory challenge^22^, the contribution of baseline inflammation or change of inflammatory markers to ketamine treatment response in depressed patients remains unclear.

A differential relationship on the basis of sex exists between inflammation, depressive symptom expression, and possibly depression treatment response^28,29^. There is evidence that inflammatory profiles differ between depressed females and males^30,31^, females are more affectively and neurally sensitive to the effects of inflammation compared to males^13,14^, and inflammation is more robustly related with depression symptom profiles in females than males in cross-sectional studies^32,33^. However, relatively little research has examined sex-specific differences in the association between inflammation and depression treatment response. One recent study found that elevated baseline C-reactive protein (CRP) was predictive of poorer antidepressant treatment outcomes in females, but not males^28^.

IL-8 is a pro-inflammatory cytokine and chemokine that is also secreted in culture in response to estradiol^34^, and may have neuroprotective properties^35–38^. A recent meta-analysis of a large number of antidepressant treatment studies examining links between inflammation and treatment response, found that lower baseline levels of IL-8, but not other biomarkers (i.e., IL-1β, IL-2, IL-4, IL-5, IL-6, IL-10, IL-12, TNF-α, IFN-γ, GM-CSF, MIP-1α, Eotaxin-1, and CRP) were associated with depression treatment response^39^, although sex differences were not systematically evaluated. Recently, we examined the association between IL-8 and response to ECT in a sample of TRD patients, and found that lower baseline levels of IL-8, as well as increases of IL-8 over ECT, were associated with treatment response in females, but not males^29^.

This study extends our prior work to a disparate treatment modality and evaluates whether IL-8 is associated with treatment response in a different sample of TRD patients undergoing open label ketamine infusion, with evaluation of sex differences (*n* = 46; 17 females). Based on our findings in ECT patients, we hypothesized that lower IL-8 at baseline as well as subsequent increases in IL-8 following ketamine treatment would relate to a favorable treatment response in females but not males. Additionally, given interest in other potential markers of inflammation in relation to depression treatment response, we also explored relationships between several other inflammatory markers including CRP, IL-6, IL-10, TNF-α, and ketamine treatment outcome.

## Patients and methods

### Participants

Subjects were depressed patients (*N* = 46; 17 females, 29 males) who were enrolled in an open label clinical trial of ketamine at the University of California, Los Angeles (UCLA; NCT02165449). Partway through the initial study offering single session ketamine infusion, the clinical trial was amended to include serial treatment for a second study recruiting the same TRD population. Only time points available in both the original and amended protocol (baseline and 24 h following one ketamine infusion) were investigated in this analysis.

This study reports on a subsample of those reported on previously for imaging analyses^40–42^, and includes participants who received an infusion of ketamine and for whom inflammatory marker data were available. This sample does not overlap with the previously evaluated TRD sample that received ECT. All procedures were approved by the UCLA Institutional Review Board. Written informed consent was obtained from all participants. Data were collected between May 2013 and March 2018.

Inclusion criteria were recurrent major depressive episode, and failure to achieve therapeutic response to at least two trials of antidepressant medication of sufficient dose used for at least 4–6 weeks each, during the current episode, as determined by the Antidepressant Treatment History Form. Recurrent major depressive disorder was diagnosed using a structured clinical interview and Diagnostic and Statistical Manual of Mental Disorders criteria. Exclusion criteria were as follows: history of alcohol or substance abuse within the past 6 months and/or dependence within the past 12 months, intellectual disability, primary psychotic disorder, metal implants (i.e. pacemakers, defibrillators, aneurysm clips, etc), neurologic illness, and serious medical illness. Specifically, patients were asked at screening if they had type 1 diabetes, were insulin dependent, or had “any other serious medical illness.” If “yes” to any of those questions, the potential participant was excluded at screening. Conditions not identified by the patient at screening as “serious” but identified during the subsequent consultation were excluded as “unstable medical conditions” if requiring active management as indicated by ongoing medication or applicable treatment adjustments to achieve control of the condition in question or which, in the opinion of the Principal Investigator, could pose a risk from ketamine exposure to the patient. For example, patients were excluded if baseline blood pressure was >140/90 and not normalized with intervention if indicated. Laboratory studies were reviewed, including liver function tests and basic metabolic panel, and patients were excluded if laboratory tests suggested organ dysfunction. Stable regimens of psychotropic medications were continued. Benzodiazepines, if prescribed, were withheld the night before ketamine treatment.

### Procedures

Participants received an open label infusion of ketamine (0.5 mg/kg infused intravenously over 40 min), with formal clinical assessments and blood sampling at baseline and post-treatment (approximately 24 h following ketamine infusion). Clinical assessment of depressive symptom severity and blood sampling for inflammatory markers were obtained at both time points. For participants with BMI ≥ 30 kg/m^2^, ketamine was dosed using adjusted body weight.

### Clinical assessment of depressive symptom severity

The 17-item Hamilton Depression Rating Scale (HAM-D)^43^ was collected at baseline and post-treatment (approximately 24 h following ketamine infusion). Though the post-treatment assessment was completed 24 h following ketamine infusion for all participants, baseline HAM-D assessment was completed a mean of 6.7 days (SD 4.3 days) in advance of the post-treatment assessment, as participants completed a lengthy day of baseline assessments and imaging studies in advance of their scheduled ketamine infusion date. For short time frames between HAM-D assessments (<1 week), assessors asked participants to assess symptoms since the last assessment instead of over the last week.

Response was defined as ≥50% reduction in HAM-D score from baseline to post-treatment^44^. Percent change in HAM-D score from baseline to end-of-treatment was also used as the continuous outcome measure.

### Assessment of inflammation

Whole blood samples were collected in EDTA tubes, chilled on wet ice, and then centrifuged at 4 °C. Plasma was harvested into multiple aliquots, and then stored in a −80 °C freezer until assay.

Plasma concentrations of pro-inflammatory cytokines IL-6, IL-8, and TNF-α, and the anti-inflammatory cytokine IL-10, were measured utilizing a Bio-Plex 200 (Luminex) instrument and a high-sensitivity multiplex immunoassay (Performance High Sensitivity Human Cytokine, R& D Systems, Minneapolis, MN). Data acquisition and analyses were performed with Bio-Plex software v4.1, and a 5-parameter logistic curve fit. As previously described, this multiplex assay has excellent intra-assay (<8% coefficient of variation [CV]) and inter-assay (11–16% CV) reproducibility^45^. Multiplex assays were performed on samples diluted 2-fold according to the manufacturer’s protocol. Plasma concentrations of CRP were determined utilizing the Human CRP Quantikine ELISA (R&D Systems) according to the manufacturer’s protocol with the following modifications: samples were diluted 500-fold, and the standard curve was extended to 0.4 ng/mL to obtain a lower limit of detection of 0.2 mg/L, taking sample dilution into account. Mean intra-assay CV was <9%, inter-assay CV was ≤15%. All cytokine assays were performed in duplicate, with all samples from a single individual tested on the same assay plate. The mean of the duplicate sample was used in all analyses.

Levels of IL-8, IL-10, and TNF-α were detectable in 100% of samples. For the small proportion (9%) of samples with IL-6 concentrations below the lower limit of detection (0.1 pg/mL), a value equal to one-half the lower limit (0.05 pg/mL) was assigned. For the small proportion (3%) of samples with CRP concentrations below the limit of detection (0.2 mg/L), a value equal to one-half the lower limit (0.1 mg/L) was assigned. No samples had CRP concentrations above the upper limit of the standard curve (>25 mg/L).

### Statistical analyses

All statistical analyses were conducted using the IBM SPSS (Version 26, IBM Corp, Armonk, New York). As cytokine and CRP data were not normally distributed, we performed a base-10 logarithmic transformation on the data prior to statistical analyses.

The sample size was determined given prior findings for ECT treatment response in depressed patients^29^ which observed a large effect (modified d-effect size = 0.97) for the three-way interaction between responder status, sex, and baseline to post-treatment changes in IL-8; this is the primary analysis in the present study. The anticipated effect was adjusted downward to a more standard *d* = 0.80 level with a total sample size target of 42 for 80% power (alpha = 0.05) assuming distributions of sex and responder status no more extreme than 2:1.

A linear regression model was used to evaluate the joint effects of responder status (i.e., responder, non-responder) and sex (i.e., females, males), on baseline concentration of IL-8. A mixed linear effects model was used to evaluate the joint effects of responder status (i.e., responder, non-responder), sex (i.e., females, males), and time (baseline, post-treatment) on IL-8 change. Clustering of repeated measurements from the same participant was modeled with a random effect of the individual. Analyses included only participants who had completed an infusion of ketamine, and had pre- and post-treatment HAM-D scores and inflammatory data available for analysis. All analyses included age and BMI as covariates, which were not missing for any subject. Exploratory analyses were completed for other inflammatory markers including IL-6, IL-10, TNF- α, and CRP, and are included as supplementary results.

Where there was evidence of a three-way interaction between responder status, sex, and time, linear regression analyses examined the relationships between change in the inflammatory marker (post-treatment minus baseline) and the continuous outcome measure, percentage change in HAM-D scores: [(post-treatment minus baseline)/baseline] × 100.

Sensitivity analyses were completed utilizing modified HAM-D scores which excluded the three sleep items, given the challenge of assessing change in sleep over short time frames. These analyses are included as supplementary results.

Significance was evaluated at an alpha level of α=0.05, two-tailed.

## Results

Table 1 summarizes patient demographic and treatment information. Thirty-seven percent of participants were females (*n* = 17). Fifty percent (*n* = 23) of participants met criteria for treatment response after a single ketamine infusion. No statistically significant differences in demographic variables or clinical severity variables were identified between females and males, nor between responders and non-responders.

**Table 1:** Baseline characteristics and treatment information for the study sample (*n* = 46).

		Sex differences (males vs females)			Group differences (responders vs non-responders)		
	Overall	Male	Female	Sex difference^a^	Responders (*n* = 23)	Non-responders (*n* = 23)	Responder difference^a^
*Demographic information*							
Sex, M/F, *n*	29/17	63%	37%		15/8	14/9	0.99
Age, mean (SD), *y*	42.3 (11.6)	40.0 (11.9)	43.5 (11.1)	0.32	42.9 (9.4)	39.7 (13.5)	0.35
BMI, mean (SD), kg/m^2^	26.2 (6.4)	26.7 (5.7)	25.4 (7.6)	0.51	26.0 (6.8)	26.5 (6.1)	0.80
Education, mean (SD), ISCED level^b^	5.6 (1.4)	5.4 (1.3)	5.9 (1.4)	0.26	5.6 (1.5)	5.5 (1.2)	0.91
*Clinical information*							
Age at depression diagnosis, mean (SD), y	21.2 (8.0)	21.1 (9.5)	21.2 (4.6)	0.99	22.2 (7.3)	20.1 (8.7)	0.40
Current episode duration, median (IQR), y	4.0 (9.0)	5.0 (14.0)	2.8 (6.5)	0.39	3.5 (6.0)	4.0 (14.0)	0.36
Lifetime illness duration, mean (SD), y	21.4 (11.0)	20.8 (11.6)	22.4 (10.1)	0.64	21.7 (9.4)	21.1 (12.6)	0.87
Unipolar/bipolar depression, *n*	41/5	25/4	16/1	0.64	18/5	23/0	0.05

^a^*p* value shown for differences evaluated with t-test, Mann–Whitney U-test, or Fisher’s exact test.

^b^International Standard Classification of Education, UNESCO Institute for Statistics: Levels range from 0 (less than primary education) to 8 (doctoral level or equivalent); level 3 is equivalent to a high school diploma^49^.

### Baseline interleukin-8 and depression treatment response

**Fig. 1:**
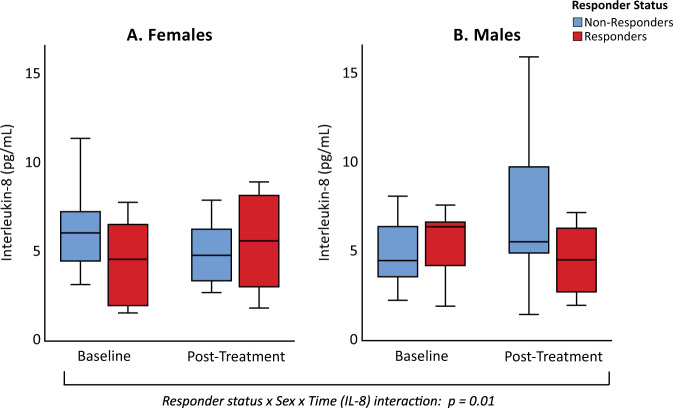
Interleukin-8 at baseline and post-treatment in responders versus non-responders, stratified by sex. Levels of IL-8 at baseline and post-treatment (approximately 24 h following ketamine infusion) are shown in female (**A**, *n* = 17) and male (**B**, *n* = 29) subjects, stratified by non-responders (blue boxes) and responders (red boxes). Baseline levels of IL-8 varied in relation to responder status and sex (responder status × sex interaction: β = −0.36, *p* = 0.096). Females, but not males, showed a statistical trend between lower baseline IL-8 levels and depression response to ketamine (females: *p* = 0.095, effect size (sr^2^) = 0.16; males: *p* = 0.96, effect size (sr^2^) <0.01). Change in levels of IL-8 from baseline to post-treatment varied in relation to responder status and sex (responder status × sex × time interaction): *F*(1,42)=6.68, *p* = 0.01.

Linear regression models were used to evaluate whether baseline level of IL-8 was associated with responder status, depending upon sex (Table 2). Lower baseline IL-8 was associated with more favorable antidepressant response to ketamine depending upon sex, although the interaction did not reach statistical significance (responder status × sex interaction: β = -0.36, *p* = 0.096), with evidence of an association in females (β = −0.41, *p* = 0.095, effect size (sr^2^) = 0.16), but not males (β= −0.01, *p* = 0.96, effect size (sr^2^) <0.01) (Fig. 1). There were no main effects of responder status (β = −0.18, *p* = 0.21) or sex (β = 0.03, *p* = 0.83) on baseline levels of IL-8. No other inflammatory markers at baseline related to responder status, or the interaction between responder status and sex (Supplementary Table 1).

**Table 2:** Interleukin-8 and HAM-D at baseline and post-treatment, in relation to responder status and sex.

	Interleukin-8 concentration [median (Q1-Q3)] and HAM-D Scores [mean (SD)]		Linear regression and linear mixed models evaluating effects of responder status and sex on IL-8 concentration		
	Baseline	Post-treatment (24 h)	Model	Standardized β coefficient or *F* value	*p* value
**IL-8, pg/mL**^**a**^ **(*n*** **=** **46)**	5.1 (3.7–6.9)	5.2 (3.0–7.1)	Linear regression model for baseline IL-8^b^:		
Non-responders (*n* = 23)	5.6 (3.8–7.1)	5.3 (3.4–8.0)	Responder status × Sex	β = −0.36	0.096
Male (*n* = 14)	4.5 (3.6–6.4)	5.6 (4.3–10.1)	Linear mixed effects model for IL-8 change from baseline to post-treatment^c^:		
Female (*n* = 9)	6.1 (4.2–9.3)	4.8 (3.4–6.4)	Time	*F*(1,42) < 0.01	0.99
Responders (*n* = 23)	5.0 (2.7–6.7)	4.6 (2.7–6.9)	Responder Status × Time	*F*(1,42) = 0.44	0.51
Male (*n* = 15)	6.4 (4.1–6.7)	4.6 (2.7–6.5)	Sex × Time	*F*(1,42) = 0.03	0.87
Female (*n* = 8)	4.6 (1.9–7.2)	5.6 (2.6–8.6)	Responder Status × Sex × Time	*F*(1,42) = 6.68	0.01
HAM-D Score (*n* = 46)	20.4 (5.1)	10.0 (6.3)			
Non-responders (*n* = 23)	19.5 (5.2)	15.2 (3.8)			
Male (*n* = 14)	18.4 (4.9)	15.3 (4.3)			
Female (*n* = 9)	21.9 (5.3)	15.7 (3.5)			
Responders (*n* = 23)	21.3 (5.1)	5.1 (3.6)			
Male (*n* = 15)	20.7 (4.6)	4.7 (3.8)			
Female (*n* = 8)	22.4 (6.1)	5.9 (3.3)			

^a^Values were transformed by base 10 logarithm before statistical analyses, but original scale medians and quartiles (Q1−Q3) are presented.

^b^Linear regression models evaluated the joint effect of responder status and sex on baseline IL-8. Analyses included BMI and age as covariates.

^c^Linear mixed effects models evaluated the joint effect of responder status, sex, and time, on change in IL-8 concentration. Analyses included BMI and age as covariates.

### Change in interleukin-8 in relation to depression treatment response

A mixed linear effects model was used to evaluate whether change in IL-8 from baseline to post-treatment was associated with responder status, depending upon sex. Levels of IL-8 changed differentially from baseline to post-treatment in relation to responder status and sex (responder status × sex × time interaction: *F*(1,42)=6.68, *p* = 0.01) (Table 2, Fig. 1). There were no significant main effects of time [*F*(1,42)<0.01, *p* = 0.99], sex [*F*(1,40)<0.01, *p* = 0.99], or responder status [*F*(1,40)=1.8, *p* = 0.19], for IL-8. In exploratory analyses, there was no evidence that other inflammatory markers changed differentially in relation to responder status and sex. Specifically, there were no main effects of time, responder status, sex, nor any significant interaction terms for levels of IL-6, IL-10, TNF- α, or CRP (Supplementary Table 1).

### Change in interleukin-8 in relation to changes in depressive symptom severity

**Fig. 2:**
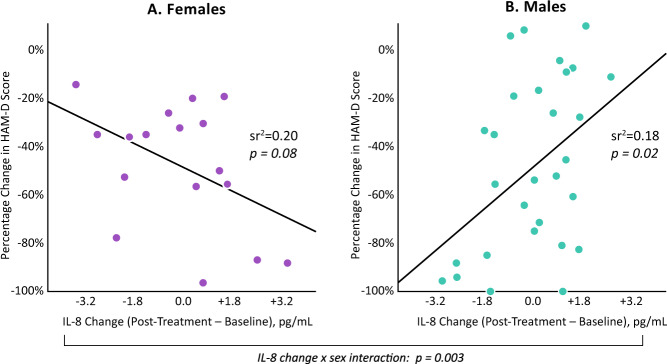
Interleukin-8 change from baseline to post-treatment associates with HAM-D percentage change, depending upon sex. Effect sizes (sr^2^) and *p* values are shown from linear regression analyses of the relationship between the change in IL-8 (post-treatment [approximately 24 h following ketamine infusion] minus baseline) and the percentage change in HAM-D score, in females (**A**) and in males (**B**). Change in IL-8 was calculated and plotted utilizing base-10 log-transformed data, but the x-axis is labeled with non-transformed values (pg/mL) for ease of interpretation. Change in IL-8 interacted with sex to predict change in HAM-D score (β = −0.63, *p* = 0.003). All analyses included age and BMI as covariates.

To further examine change in IL-8 and depression response to ketamine, percentage change in HAM-D score was calculated from baseline to post-treatment, as a continuous measurement of depression response, given the limited statistical power of the categorical outcome (i.e., responder status). Linear regression analysis identified associations between change in IL-8 from baseline to post-treatment and percentage change in HAM-D, depending upon sex (IL-8 change × sex interaction: β = −0.63, *p* = 0.003). Analyses stratified by sex showed that increasing IL-8 was associated with decreasing HAM-D score in females (β = −0.45, *p* = 0.08, effect size (sr^2^)=0.20), while the inverse was found in males (β = 0.43, *p* = 0.02, effect size (sr^2^)=0.18) (Fig. 2).

## Discussion

Here we report that an increase in IL-8 from baseline to post-treatment (24 h following ketamine infusion) was associated with a favorable depression treatment response to ketamine infusion in females, whereas increase in IL-8 was associated with an unfavorable treatment response in males. Further, lower baseline IL-8 tended to associate with greater likelihood of depression treatment response to ketamine among females, but not males. Exploratory analyses of IL-6, IL-10, TNF-α, and CRP, did not identify relationships between levels of these other inflammatory markers and ketamine treatment outcome.

It is unknown why the relationship between changing IL-8 and depression scores in response to ketamine might move in opposite directions between females versus males. However, estradiol has been found to increase secretion of IL-8 by immature dendritic cells in culture^34^; thus, there may be a relationship between sex hormones and IL-8. Of interest, pre-clinical studies have shown that females are more responsive to the behavioral effects of ketamine, and that this greater sensitivity is dependent upon female sex hormones^46–48^. It is unknown whether the inflammatory milieu may play any role in this relationship.

We have previously identified relationships between IL-8 and depression outcome in patients treated with ECT that are similar to the current findings. That is, lower baseline IL-8, as well as subsequent increase in IL-8 over a course of ECT were associated with a more favorable depression treatment response in females, but not males^29^. The association between IL-8 and treatment response to ketamine, along with treatment response to ECT, depending on sex, suggest that possible sex-specific mechanisms underlie the neural and/or behavioral effects of IL-8, although interrogation of these mechanisms will require larger samples, along with evaluation of affective response mechanisms such as reward processes.

Interestingly, in a meta-analysis of depressed patients treated with antidepressant medication, the only identified baseline inflammatory predictor of subsequent treatment response was IL-8^39^. Similar to findings among females in our prior ECT and present ketamine studies, responders to antidepressant treatment (without sex-specific evaluation) had lower baseline IL-8^39^. However, IL-8 change did not emerge as a correlate of treatment response in the antidepressant meta-analysis^39^. In the current study of TRD subjects treated with ketamine, and in our prior study of TRD subjects treated with ECT, no relationship between IL-8 change and depressive symptom change would have emerged had we analyzed the data without regard to sex. It thus remains plausible that similar sex-specific inverse relationships between IL-8 change and depressive change may exist in response to antidepressants, but this issue has not been systematically evaluated.

We did not find evidence of significant relationships between ketamine treatment outcome and IL-6, IL-10, TNF-α, and CRP levels, including when evaluated according to sex. While some studies have suggested the possibility that higher inflammation at baseline may predict better treatment response to ketamine^24,25^, we did not find evidence of this relationship in the current study, nor have two additional studies of inflammatory markers and ketamine treatment outcome^26,27^. Furthermore, it does not appear that a single ketamine infusion alters levels of these other inflammatory markers in relation to responder status or sex. It is possible that larger studies are needed to detect such relationships or that other measures of inflammation (e.g. upstream measures of transcription factor activation or central nervous system measures of inflammation) may be more sensitive.

There are several study limitations. Stable doses of psychotropic medications were continued, and it is unknown whether certain psychotropic medications may impact inflammatory markers or treatment response to ketamine. Additionally, detailed data regarding non-psychiatric medication use were not available; though serious medical conditions were excluded (including those that would require systemic corticosteroids or traditional immune modulating agents, etc), the lack of data regarding the use of medications that may impact the immune system, e.g. non-steroidal anti-inflammatory drugs, is a limitation of the current study. Also, as a naturalistic treatment study evaluating biomarkers of response to ketamine, there was no randomized arm with which to compare inflammatory or depressive changes. Further, we did not assay for sex hormones, and we do not have central or upstream (e.g. transcriptional) measures of inflammation, both of which would be a strength of future work in this area. Also, females represented only 37% of the sample evaluated in this study and power may have been too low to provide a complete picture. Despite the fact that depression is more prevalent in females, a greater number of males responded to targeted advertisements and/or were eligible for the current study. Replication in larger samples with a greater representation of females is highly recommended; at this point, current findings should be interpreted as preliminary, with a need for follow-up in larger samples with greater balance in the number of males and females.

This report provides novel evidence that lower baseline IL-8 and subsequent IL-8 increase may be uniquely related to depression improvement in females treated with ketamine, and further, that IL-8 decrease may associate with depression improvement in males. Together with previous work demonstrating similar findings among patients treated with ECT, this sex-specific finding for IL-8 across two disparate treatment modalities suggests a need for further sex-specific investigation of the role of IL-8 in depression pathophysiology and treatment responsiveness, with attention to potential behavioral and/or neural mechanisms that may underlie these relationships.

## Supplementary information

SUPPLEMENTARY RESULTS

## References

[1] Friedrich MJ (2017). Depression is the leading cause of disability around the world. JAMA.

[2] Bromet E (2011). Cross-national epidemiology of DSM-IV major depressive episode. BMC Med..

[3] Rush AJ (2006). Acute and longer-term outcomes in depressed outpatients requiring one or several treatment steps: a STAR*D report. Am. J. Psychiatry.

[4] Gimeno D (2009). Associations of C-reactive protein and interleukin-6 with cognitive symptoms of depression: 12-year follow-up of the Whitehall II study. Psychol. Med..

[5] Au B, Smith KJ, Gariepy G, Schmitz N (2015). The longitudinal associations between C-reactive protein and depressive symptoms: evidence from the English Longitudinal Study of Ageing (ELSA). Int. J. Geriatr. Psychiatry.

[6] Reichenberg A (2001). Cytokine-associated emotional and cognitive disturbances in humans. Arch. Gen. Psychiatry.

[7] Eisenberger NI (2010). Inflammation-induced anhedonia: endotoxin reduces ventral striatum responses to reward. Biol. Psychiatry.

[8] Moieni M (2015). Sex differences in depressive and socioemotional responses to an inflammatory challenge: implications for sex differences in depression. Neuropsychopharmacology.

[9] Capuron L (2002). Neurobehavioral effects of interferon-alpha in cancer patients: phenomenology and paroxetine responsiveness of symptom dimensions. Neuropsychopharmacology.

[10] Bonaccorso S (2002). Increased depressive ratings in patients with hepatitis C receiving interferon-alpha-based immunotherapy are related to interferon-alpha-induced changes in the serotonergic system. J. Clin. Psychopharmacol..

[11] Dowlati Y (2010). A meta-analysis of cytokines in major depression. Biol. Psychiatry.

[12] Haapakoski R, Mathieu J, Ebmeier KP, Alenius H, Kivimaki M (2015). Cumulative meta-analysis of interleukins 6 and 1beta, tumour necrosis factor alpha and C-reactive protein in patients with major depressive disorder. Brain Behav. Immunol..

[13] Moieni M., et al. Sex differences in the relationship between inflammation and reward sensitivity: a randomized controlled trial of endotoxin. *Biol. Psychiatry: Cogn. Neurosci. Neuroimaging* 4, 619–626 (2019). 10.1016/j.bpsc.2019.03.010 PMC6612452 31103547

[14] Udina M (2012). Interferon-induced depression in chronic hepatitis C: a systematic review and meta-analysis. J. Clin. Psychiatry.

[15] Eller T, Vasar V, Shlik J, Maron E (2008). Pro-inflammatory cytokines and treatment response to escitalopram in major depressive disorder. Prog. Neuropsychopharmacol. Biol. Psychiatry.

[16] Lanquillon S, Krieg JC, Bening-Abu-Shach U, Vedder H (2000). Cytokine production and treatment response in major depressive disorder. Neuropsychopharmacology.

[17] Chang HH (2012). Treatment response and cognitive impairment in major depression: association with C-reactive protein. Brain Behav. Immun..

[18] Uher, R. et al. An inflammatory biomarker as a differential predictor of outcome of depression treatment with escitalopram and nortriptyline. *Am. J. Psychiatry* **171**, 1278–1286 (2014). 10.1176/appi.ajp.2014.14010094 25017001

[19] Amitai M (2016). The relationship between plasma cytokine levels and response to selective serotonin reuptake inhibitor treatment in children and adolescents with depression and/or anxiety disorders. J. Child Adolesc. Psychopharmacol..

[20] Kruse, J. L., et al. Inflammation and improvement of depression following electroconvulsive therapy in treatment-resistant depression. *J. Clin. Psychiatry* **79**, 17m11597 (2018). 10.4088/JCP.17m11597 PMC6013272 29489077

[21] Carlier A (2019). Inflammation and remission in older patients with depression treated with electroconvulsive therapy; findings from the MODECT study✰. J. Affect. Disord..

[22] Walker AK (2013). NMDA receptor blockade by ketamine abrogates lipopolysaccharide-induced depressive-like behavior in C57BL/6J mice. Neuropsychopharmacology.

[23] Miller AH, Raison CL (2016). The role of inflammation in depression: from evolutionary imperative to modern treatment target. Nat. Rev. Immunol..

[24] Yang J-j (2015). Serum interleukin-6 is a predictive biomarker for ketamine’s antidepressant effect in treatment-resistant patients with major depression. Biol. Psychiatry.

[25] Machado-Vieira R., et al. The role of adipokines in the rapid antidepressant effects of ketamine. *Mol. Psychiatry* **22**, 127–133 (2016). 10.1038/mp.2016.36 PMC5112162 27046644

[26] Kiraly DD (2017). Altered peripheral immune profiles in treatment-resistant depression: response to ketamine and prediction of treatment outcome. Transl. Psychiatry.

[27] Park M (2017). Change in cytokine levels is not associated with rapid antidepressant response to ketamine in treatment-resistant depression. J. Psychiatr. Res..

[28] Jha MK (2019). Sex differences in the association of baseline c-reactive protein (CRP) and acute-phase treatment outcomes in major depressive disorder: Findings from the EMBARC study. J. Psychiatr. Res..

[29] Kruse JL (2020). Inflammation and depression treatment response to electroconvulsive therapy: Sex-specific role of interleukin-8. Brain Behav. Immun..

[30] Rainville JR, Hodes GE (2019). Inflaming sex differences in mood disorders. Neuropsychopharmacology.

[31] Labonte B (2017). Sex-specific transcriptional signatures in human depression. Nat. Med..

[32] Kohler-Forsberg O (2017). Association between C-reactive protein (CRP) with depression symptom severity and specific depressive symptoms in major depression. Brain Behav. Immun..

[33] Felger, J. C. et al. What does plasma CRP tell us about peripheral and central inflammation in depression? *Mol. Psychiatry* **25**, 1301–1311 (2018). 10.1038/s41380-018-0096-3 PMC6291384 29895893

[34] Bengtsson AK, Ryan EJ, Giordano D, Magaletti DM, Clark EA (2004). 17beta-estradiol (E2) modulates cytokine and chemokine expression in human monocyte-derived dendritic cells. Blood.

[35] Puma C, Danik M, Quirion R, Ramon F, Williams S (2001). The chemokine interleukin-8 acutely reduces Ca(2+) currents in identified cholinergic septal neurons expressing CXCR1 and CXCR2 receptor mRNAs. J. Neurochem..

[36] Giovannelli A (1998). CXC chemokines interleukin-8 (IL-8) and growth-related gene product alpha (GROalpha) modulate Purkinje neuron activity in mouse cerebellum. J. Neuroimmunol..

[37] Saas, P., et al. A self-defence mechanism of astrocytes against Fas-mediated death involving interleukin-8 and CXCR2. *Neuroreport* **13**, 1921–1924 (2002). 10.1097/00001756-200210280-00018 12395092

[38] Araujo DM, Cotman CW (1993). Trophic effects of interleukin-4, -7 and -8 on hippocampal neuronal cultures: potential involvement of glial-derived factors. Brain Res..

[39] Liu JJ (2019). Peripheral cytokine levels and response to antidepressant treatment in depression: a systematic review and meta-analysis. Mol. Psychiatry.

[40] Loureiro JRA (2020). Modulation of amygdala reactivity following rapidly acting interventions for major depression. Hum. Brain Mapp..

[41] Sahib AK (2020). Single and repeated ketamine treatment induces perfusion changes in sensory and limbic networks in major depressive disorder. Eur. Neuropsychopharmacol..

[42] Vasavada MM (2016). Structural connectivity and response to ketamine therapy in major depression: a preliminary study. J. Affect. Disord..

[43] Hamilton M (1960). A rating scale for depression. J. Neurol. Neurosurg. Psychiatry.

[44] Gelenberg, A. F. M., Markowitz, J. & Rosenbaum, J. *Practice Guideline for the Treatment of Patients with Major Depressive Disorder* (American Psychiatric Association, 2010).

[45] Epstein MM (2013). Temporal stability of serum concentrations of cytokines and soluble receptors measured across two years in low-risk HIV-seronegative men. Cancer Epidemiol. Biomark. Prev..

[46] Dossat AM, Wright KN, Strong CE, Kabbaj M (2018). Behavioral and biochemical sensitivity to low doses of ketamine: Influence of estrous cycle in C57BL/6 mice. Neuropharmacology.

[47] Saland SK, Schoepfer KJ, Kabbaj M (2016). Hedonic sensitivity to low-dose ketamine is modulated by gonadal hormones in a sex-dependent manner. Sci. Rep..

[48] Carrier N, Kabbaj M (2013). Sex differences in the antidepressant-like effects of ketamine. Neuropharmacology.

[49] International Standard Classification of Education (ISCED). Montreal, Canada, UNESCO Institute for Statistics (2012). http://uis.unesco.org/sites/default/files/documents/international-standard-classification-of-education-isced-2011-en.pdf.

