# Supplementary material for: Depression treatment response to ketamine: sex-specific role of interleukin-8, but not other inflammatory markers
Source: Transl Psychiatry. 2021 Mar 15;11:167. doi: 10.1038/s41398-021-01268-z (PMC7960960; doi:10.1038/s41398-021-01268-z)
Supplement: Supplementary file 1 — SUPPLEMENTARY RESULTS [file 41398_2021_1268_MOESM1_ESM.docx]

**SUPPLEMENTARY RESULTS**

Sensitivity analyses utilizing modified HAM-D scores (excluding the three sleep items from both baseline and post-treatment HAM-D assessments) were completed, given the challenge of assessing change in sleep over short time frames. Forty-six percent (n=21) of participants met criteria for treatment response utilizing modified HAM-D scores, after a single ketamine infusion.

*Baseline Interleukin-8 and Depression Treatment Response*

Linear regression models were used to evaluate whether baseline level of IL-8 was associated with responder status, depending upon sex. There were no substantive differences in results utilizing modified HAM-D scores (excluding sleep items) compared to actual HAM-D scores. Lower baseline IL-8 was associated with more favorable antidepressant response to ketamine depending upon sex, although the interaction did not reach statistical significance (responder status x sex interaction: β = -0.44, p=0.053), with evidence of an association in females (β = -0.41, p=0.093, effect size (sr^2^) = 0.16), but not males (β= 0.12, p=0.53, effect size (sr^2^) = 0.01).

*Change in Interleukin-8 in Relation to Depression Treatment Response*

A mixed linear effects model was used to evaluate whether change in IL-8 from baseline to post-treatment was associated with responder status, depending upon sex. There were no substantive differences in results utilizing modified HAM-D scores (excluding sleep items) compared to actual HAM-D scores. Levels of IL-8 changed differentially from baseline to post-treatment in relation to responder status and sex (responder status x sex x time interaction: F(1,42)=6.32, p=0.02). There were no significant main effects of time [F(1,42)<0.02, p=0.89], sex [F(1,40)<0.01, p=0.97], or responder status [F(1,40)=0.78, p=0.38], for IL-8.

*Change in Interleukin-8 in Relation to Changes in Depressive Symptom Severity*

To further examine change in IL-8 and depression response to ketamine, percentage change in HAM-D score was calculated from baseline to post-treatment, as a continuous measurement of depression response, given the limited statistical power of the categorical outcome (i.e., responder status). There were no substantive differences in results utilizing modified HAM-D scores (excluding sleep items) compared to actual HAM-D scores. Linear regression analysis identified associations between change in IL-8 from baseline to post-treatment and percentage change in HAM-D, depending upon sex (IL-8 change x sex interaction: β= -0.67, p=0.001). Analyses stratified by sex showed that increasing IL-8 was associated with decreasing HAM-D score in females (β= -0.48, p=0.06, effect size (sr^2^)=0.23), while the inverse was found in males (β= 0.43, p=0.02, effect size (sr^2^)=0.18).

**Other Inflammatory Markers and Depression Response to Ketamine**

Relationships between treatment response, sex, and four additional immune markers (IL-6, IL-10, TNF-a, and CRP) were explored. There were no main effects of time, responder status, sex, nor any significant interaction terms identified for IL-6, IL-10, TNF-a, or CRP concentrations. (Supplementary Table 1).

| **Supplementary Table 1.**  **Other Immune Markers at Baseline and Post-Treatment, in Relation to Responder Status and Sex** | | | | | |
| --- | --- | --- | --- | --- | --- |
|  | **Immune Marker Concentration [median (Q1-Q3)]** | | **Models Evaluating Effects of Time, Responder Status (Group), and Sex on Immune Marker Concentration** | | |
|  |  |  |  |  |  |
|  | **Baseline** | **Post-Treatment (24H)** | **Model** | **Standardized β Coefficient or F value** | ***p* value** |
| **IL-6, pg/mL^*^ (n=46)** | 1.1 (0.5-1.6) | 0.9 (0.4-1.6) | **Linear Regression model for baseline IL-6^1^:** | | |
| **Non-responders (n=23)** | 1.2 (0.5-1.6) | 0.9 (0.4-1.6) | Responder Status x Sex | β = 0.11 | 0.62 |
| Male (n=14) | 1.2 (0.5-1.7) | 0.9 (0.5-1.7) | **Linear mixed effects model for IL-6 change from baseline to post-treatment^2^:** | | |
| Female (n=9) | 1.2 (0.3-1.5) | 0.9 (0.1-1.8) | Time | F(1,42)=1.88 | 0.18 |
| **Responders (n=23)** | 0.9 (0.5-1.3) | 0.9 (0.4-1.2) | Responder Status x Time | F(1,42)=0.11 | 0.74 |
| Male (n=15) | 1.2 (0.7-1.6) | 0.9 (0.4-1.2) | Sex x Time | F(1,42) <0.01 | 0.96 |
| Female (n=8) | 0.8 (0.4-1.0) | 0.8 (0.3-1.4) | Responder Status x Sex x Time | F(1,42)=0.09 | 0.77 |
| **IL-10, pg/mL^*^ (n=46)** | 0.5 (0.3-0.6) | 0.5 (0.3-0.7) | **Linear Regression model for baseline IL-10^1^:** | | |
| **Non-responders (n=23)** | 0.5 (0.3-0.6) | 0.5 (0.3-0.7) | Responder Status x Sex | β = 0.17 | 0.48 |
| Male (n=14) | 0.5 (0.3-0.7) | 0.5 (0.3-0.7) | **Linear mixed effects model for IL-10 change from baseline to post-treatment^2^:** | | |
| Female (n=9) | 0.5 (0.4-0.7) | 0.5 (0.3-0.7) | Time | F(1,42)=0.01 | 0.91 |
| **Responders (n=23)** | 0.4 (0.3-0.6) | 0.5 (0.3-0.7) | Responder Status x Time | F(1,42)=0.55 | 0.47 |
| Male (n=15) | 0.3 (0.3-0.6) | 0.4 (0.3-0.8) | Sex x Time | F(1,42)=0.56 | 0.46 |
| Female (n=8) | 0.5 (0.4-0.7) | 0.5 (0.3-0.7) | Responder Status x Sex x Time | F(1,42)=0.02 | 0.88 |
| **TNF-α, pg/mL^*^ (n=46)** | 5.7 (4.3-7.5) | 6.0 (4.8-7.0) | **Linear Regression model for baseline TNF-α^1^:** | | |
| **Non-responders (n=23)** | 6.1 (4.5-7.7) | 6.1 (5.2-7.8) | Responder Status x Sex | β = -0.05 | 0.85 |
| Male (n=14) | 7.3 (4.6-8.9) | 7.1 (5.5-8.7) | **Linear mixed effects model for TNF-α change from baseline to post-treatment^2^:** | | |
| Female (n=9) | 5.2 (4.2-6.2) | 6.0 (2.4-6.2) | Time | F(1,42)=0.10 | 0.76 |
| **Responders (n=23)** | 5.7 (3.9-6.7) | 5.4 (4.5-6.8) | Responder Status x Time | F(1,42)=0.37 | 0.55 |
| Male (n=15) | 6.2 (5.0-7.9) | 6.4 (5.0-7.0) | Sex x Time | F(1,42)=2.56 | 0.12 |
| Female (n=8) | 4.8 (3.9-5.7) | 4.7 (3.6-5.9) | Responder Status x Sex x Time | F(1,42)=1.24 | 0.27 |
| **CRP, mg/L^*^ (n=46)** | 0.9 (0.5-1.8) | 0.7 (0.3-1.6) | **Linear Regression model for baseline CRP^1^:** | | |
| **Non-responders (n=23)** | 1.0 (0.6-2.1) | 0.8 (0.3-2.2) | Responder Status x Sex | β = -0.33 | 0.15 |
| Male (n=14) | 0.7 (0.3-1.7) | 0.5 (0.3-1.8) | **Linear mixed effects model for CRP change from baseline to post-treatment^2^:** | | |
| Female (n=9) | 1.3 (0.7-3.6) | 0.9 (0.4-2.4) | Time | F(1,42)=2.86 | 0.10 |
| **Responders (n=23)** | 0.6 (0.5-1.4) | 0.7 (0.3-1.4) | Responder Status x Time | F(1,42)=1.74 | 0.19 |
| Male (n=15) | 0.7 (0.5-1.8) | 0.6 (0.3-1.4) | Sex x Time | F(1,42) <0.01 | 0.96 |
| Female (n=8) | 0.8 (0.5-1.4) | 0.8 (0.5-1.4) | Responder Status x Sex x Time | F(1,42)=1.70 | 0.20 |
| *Values were transformed by base 10 logarithm before statistical analyses, but original scale medians and quartiles (Q1-Q3) are presented. | | | | |  |
| ^1^Linear regression models evaluated the joint effect of responder status and sex on the baseline concentration of the inflammatory marker. All analyses included BMI and age as covariates. | | | | | |
| ^2^Linear mixed effects models evaluated the joint effect of responder status, sex, and time, on change in inflammatory markers over treatment. All analyses included BMI and age as covariates. | | | | | |
